# Editorial: Placental dysfunction in pregnancy: endocrine and metabolic mechanisms in preeclampsia, FGR, diabetes, and hypertension

**DOI:** 10.3389/fendo.2025.1756523

**Published:** 2025-12-19

**Authors:** Sruthi Alahari, Leonardo Ermini

**Affiliations:** 1 Lunenfeld-Tanenbaum Research Institute, Mount Sinai Hospital, University of Toronto, Toronto, ON, Canada; 2 Department of Life Sciences, Universita degli Studi di Siena, Siena, Italy

**Keywords:** adverse pregnancy, fetal growth restriction (FGR), gestational diabètes, placenta, preeclampsia, pregnancy

Pregnancy complications such as preeclampsia, fetal growth restriction (FGR), gestational diabetes, and hypertensive disorders remain leading causes of maternal and perinatal morbidity worldwide, underscoring the urgent need to better understand the biological mechanisms that drive these conditions. Central to these syndromes is the placenta, a dynamic endocrine and metabolic organ that orchestrates maternal–fetal exchange, hormonal communication, immune modulation, and vascular adaptation. Disruptions in placental signaling can impair these tightly regulated processes and contribute to poor pregnancy outcomes. This Research Topic, *Placental Dysfunction in Pregnancy: Endocrine and Metabolic Mechanisms in Preeclampsia, FGR, Diabetes, and Hypertension*, brings together nine original research articles that collectively advance our mechanistic understanding of placental dysfunction and highlight emerging biomarkers and molecular pathways with diagnostic and therapeutic potential. Together, these studies address key knowledge gaps in how endocrine, metabolic, vascular, and immunologic disturbances converge to produce clinically significant pregnancy disorders.

A central theme across several contributions is the identification of early circulating biomarkers that reflect underlying placental pathology. In a large prospective cohort of nearly 6,000 pregnancies, Li et al. demonstrated that maternal serum placental growth factor (PlGF) measured in early gestation is strongly and inversely associated with the risk of preeclampsia and small-for-gestational-age neonates, supporting its value for gestational age–specific risk stratification across modes of conception. Complementing these angiogenic insights, Wei et al. analyzed endocrine trajectories and showed that women with a “high and steadily rising” estradiol trajectory during early pregnancy had a significantly lower risk of early miscarriage compared with those with low and slowly increasing levels, underscoring the prognostic significance of dynamic hormonal patterns rather than isolated measurements. In a related retrospective analysis from the same group, repeated-measures modeling of progesterone identified decline thresholds that were strongly associated with early pregnancy loss, demonstrating a dose–response relationship between the number of threshold crossings and miscarriage risk. These findings highlight the clinical relevance of monitoring progesterone changes over time for early risk assessment (Wei et al.). Taken together, the PlGF, estradiol, and progesterone studies illustrate how dynamic hormonal and angiogenic profiling may open earlier diagnostic windows for placental dysfunction.

Another major focus of this Research Topic is the molecular and cellular regulation of placental function, including circadian signaling, translational control, and mitotic fidelity. Venegas et al. characterized circadian oscillations within human placental explants and identified rhythmic expression patterns of BMAL1, PER genes, and the cell-cycle regulator WEE1. Intriguingly, exogenous melatonin suppressed these oscillations without altering the placenta’s endogenous melatonin production, suggesting a selective modulatory effect on the placental molecular clock and its links to trophoblast proliferation. Complementing this temporal dimension, Chen et al. applied explainable machine learning to integrated placental transcriptomic datasets and revealed dysregulated ribosome biogenesis as a central pathway in preeclampsia. Their model identified six predictive biomarkers: GLUL, DDX28, NCL, RIOK1, SUV39H1, and RRS1, with strong diagnostic performance and mechanistic associations with immune microenvironment alterations. This systems-level approach underscores the potential of interpretable artificial intelligence for identifying clinically meaningful biomarkers grounded in underlying biology. Together, these molecular studies highlight the importance of cellular homeostasis—including circadian regulation, translational machinery, and cell-cycle fidelity—in the pathogenesis of placental dysfunction.

Translational applications are further strengthened by the identification of non-invasive molecular indicators of placental pathology. Andrieu et al. reported that circulating dual specificity phosphatase 1 (DUSP1) is elevated at the time of preeclampsia diagnosis but normalizes postpartum, emphasizing its potential as a disease-specific and temporally resolved biomarker. Their work suggests that DUSP1 captures an aspect of active placental dysfunction and merits validation in prospective cohorts. Such markers may help distinguish current disease activity from residual risk, advancing precision monitoring strategies in pregnancy.

The downstream neonatal consequences of impaired placental function are highlighted in the work by Huang et al., who examined platelet indices in small-for-gestational-age preterm infants born to mothers with or without preeclampsia. Although neonates of preeclamptic pregnancies exhibited lower platelet counts on Day 7, the overall pattern suggested that SGA status itself may be the stronger determinant of platelet abnormalities, emphasizing the enduring hematologic effects of restricted fetal growth. In a complementary population-driven perspective from Ethiopia, Tadese et al. reported on perinatal outcomes associated with placental abruption and found that nearly 40% of affected pregnancies resulted in adverse neonatal outcomes. Severity of abruption and preterm presentation emerged as dominant predictors, highlighting the need for early recognition, patient-centered counseling, and context-specific interventions to mitigate risk in resource-limited settings. Together, these neonatal studies underscore the lasting clinical impact of placental pathology on infant health trajectories.

Finally, the developmental implications of maternal metabolic disease are illustrated by Valle-Bautista et al., who profiled early corticogenesis in embryos of diabetic dams. Their transcriptomic analysis revealed perturbations in mitotic regulation, microtubule organization, and chromosome segregation, with upregulation of AURKB and NUMA1 and corresponding structural abnormalities in neural stem cell mitosis. These alterations suggest an accelerated shift toward neurogenesis that may deplete progenitor pools and compromise cortical development, offering mechanistic insight into the neurodevelopmental vulnerabilities observed in offspring of diabetic mothers.

Together, the articles in this Research Topic offer a multidimensional exploration of placental dysfunction, spanning endocrine trajectories, molecular clocks, ribosome biology, immune contexture, clinical biomarkers, developmental pathways, and neonatal outcomes. They collectively reinforce the concept that pregnancy complications arise from a complex interplay of metabolic, vascular, immunologic, and endocrine signals that converge on placental health. As placental biology increasingly integrates multi-omics, longitudinal biomarker profiling, advanced computational modeling, and translational clinical research, future studies will be poised to identify earlier diagnostic windows and test mechanism-based therapies aimed at restoring placental function. These advances are essential for reducing the global burden of preeclampsia, FGR, gestational diabetes, and hypertensive disorders, and for improving the lifelong health trajectories of mothers and their children.

We thank all authors, reviewers, and Topic Editors for their contributions to this important field.

